# AI-orchestrated microglial sculptors: pioneering neural homeostasis restoration in 2025

**DOI:** 10.1097/MS9.0000000000003785

**Published:** 2025-08-28

**Authors:** Muhammad Talha, Maliha Khalid, Aminath Waafira

**Affiliations:** a Department of Medicine, King Edward Medical University, Lahore, Pakistan; b Department of Medicine, Jinnah Sindh Medical University, Karachi, Pakistan; c Department of Medicine, The Maldives National University, Malé, Maldives

**Keywords:** artificial intelligence (AI), microglia, neuroinflammation

## Abstract

The rising global prevalence of neuroinflammatory diseases poses a formidable public health challenge. Central to these pathologies are microglia, the innate immune sentinels of the central nervous system, whose dysregulation contributes significantly to neurodegeneration. In 2025, the convergence of artificial intelligence (AI) and neurobiology is catalyzing a paradigm shift in the restoration of neural homeostasis. This paper presents a forward-looking synthesis of how AI-driven platforms are enabling high-throughput analysis of multi-omics data to identify actionable microglial targets such as TREM2 and CSF1R for therapeutic modulation. Coupled with CRISPR/Cas9 gene editing, AI orchestrates the reprogramming of microglia toward neuroprotective phenotypes, thereby mitigating neuroinflammation and facilitating cognitive recovery in preclinical models. While promising, challenges remain: addressing microglial heterogeneity, establishing reliable biomarkers, ensuring long-term safety, and overcoming ethical and regulatory barriers. This article advocates for multidisciplinary collaboration, infrastructure reform, and equitable policy frameworks to advance AI-orchestrated microglial therapies from research to clinical application, marking a transformative stride in precision neurology.

*To the Editor,*

The rising global incidence of neuroinflammatory diseases such as Alzheimer’s disease, Parkinson’s disease, and multiple sclerosis represents a significant public health challenge, afflicting millions while putting additional strain on healthcare resources across the world. Microglia, the resident immune cells in the CNS, are important for maintaining neural homeostasis and regulating neuroinflammation, but their dysregulation leads to disease progression ^[1]^. In the past few years, advances in artificial intelligence have helped to develop novel strategies for therapeutic intervention, aiming to either reprogram microglial cells to restore CNS balance or stop neurodegenerative processes, an emergent hypothesis caught up in the momentum of 2025.

Microglia are extremely heterogeneous and multifunctional, thus swinging on a pendulum between the neuroprotective spectrum and the neurotoxic mode of action. Sustained microglial activation sets into motion the barrage of neuroinflammation, which in turn adds to neuronal insult and cognitive decline. Pharmacological intervention thus far has rendered little success due to the intricate and heterogeneous anatomy and demeanor of microglial signaling. The present rise of AI platforms permits integration and analysis of a vast amount of multi-omics data and patient-specific biomarkers to pinpoint specific molecular targets. Here, AI was utilized to explore microglial receptors such as TREM2 and CSF1R as promising drug targets that modulate microglial function for drug repurposing or identifying newer ones with better specificity and efficacy ^[2]^.

Coalescing AI with gene editing technologies, such as CRISPR/Cas9, promises in vivo microglial reprogramming like no other opportunity. Engineered microglia can then be challenged to adopt neuroprotective phenotypes, allowing the clearance of insidious structures such as amyloid-beta and the inhibition of pro-inflammatory cytokine production, by targeting genes involved in neuroinflammatory pathways. Preclinical studies show that AI-mediated microglial modulation improves cognition and reduces neurodegeneration in animal models, signifying the therapeutic promise of this approach^[3]^. Yet, various gaps in this area still need to be closed for clinical translation: gene-edited microglia must be safe in the long term, intra-microglia heterogeneity must be overcome, and methods need to be developed for establishing reliable biomarkers that can be used to monitor therapeutic responses. Ethical and regulatory issues about AI-mediated gene editing also must be considered. A multidisciplinary approach needs to be done with neuroscientists, immunologists, bioinformaticians, and clinicians to improve the effectiveness of these therapies in different patient populations and their validation^[4]^.

Furthermore, robust infrastructure and training would enhance the path for the integration of AI-orchestrated microglial therapies into clinical settings. Healthcare systems should emphasize AI literacy and lay down interprofessional educational programs, so that clinicians have the know-how to apply precision neurology. Policies must be there to facilitate equal access pathways to these advanced therapies for the benefit of all affected populations. Research grants and public-private partnerships would greatly expedite this promising innovation’s transition from bench to bedside^[5]^. Our work is in line with the TITAN Guidelines on the need for transparency in AI use in healthcare^[6]^.

To conclude, AI-guided microglial reprogramming provides a paradigm shift to restoring neural homeostasis and fighting neuroinflammatory diseases. The coordinated efforts of researchers and educators in various fields, as well as policymakers, are needed to ensure these therapies achieve their utmost potential in alleviating the global burden of neurodegenerative disorders

## Data Availability

None.
